# 

**Published:** 1915-09-04

**Authors:** 


### Tropical Medicine at Liverpool and Edinburgh University.

The Liverpool School of Tropical Medicine is carried on in conjunction with the University of Liverpool, the Royal Southern Hospital, and the Royal Infirmary, for the purposes of research and clinical teaching respectively. Special wards at the hospitals have been set apart for the reception and treatment of tropical diseases, and facilities for investigation and study are given in the Johnston Laboratories of the University. The fee for a full course (lasting thirteen weeks) is 13 guineas. A short course, specially arranged for advanced instruction, is also held during June (fee 4 guineas). The new laboratories will be opened early next year.

The University of Edinburgh grants a Diploma in Tropical Medicine and Hygiene. The examinations are held in December and June. The fees for the first and any subsequent appearance are: Practical bacteriology, £1 1s.; diseases of tropical climates, £1 1s.; tropical hygiene, £1 1s.; tropical clinical medicine, £1 1s.; total, £4 4s.

The University also grants a Diploma in Psychiatry. The fees for the first and second examinations are £5 5s. each. The first examination comprises anatomy, physiology, pathology, and bacteriology; the second examination, psychology (including experimental psychology), clinical neurology and psychiatry (systematic and clinical). The examinations will be held in March and June.

**RATES OF SUBSCRIPTION: Twelve months, 6/6; Six months, 4/-; Three months, 2/-, post free to any address in the United Kingdom. Abroad, Twelve months, 8/8; Six months, 5/-; Three months, 2/6, post free.—Address The Subscription Dept., "THE HOSPITAL," The Hospital Building, 28/29 Southampton Street, Strand, London, W.C.**

